# Sphingosine-1-phosphate promotes PDGF-dependent endothelial progenitor cell angiogenesis in human chondrosarcoma cells

**DOI:** 10.18632/aging.102508

**Published:** 2019-12-06

**Authors:** Chao-Qun Wang, Chih-Yang Lin, Yuan-Li Huang, Shih-Wei Wang, Yan Wang, Bi-Fei Huang, Yu-Wei Lai, Shun-Long Weng, Yi-Chin Fong, Chih-Hsin Tang, Zhong Lv

**Affiliations:** 1 Department of Pathology, Affiliated Dongyang Hospital of Wenzhou Medical University, Dongyang, Zhejiang, China; 2 Department of Medicine, Mackay Medical College, New Taipei, Taiwan; 3 Department of Biotechnology, College of Health Science, Asia University, Taichung, Taiwan; 4 Graduate Institute of Natural Products, College of Pharmacy, Kaohsiung Medical University, Kaohsiung, Taiwan; 5 Department of Medical Oncology, Affiliated Dongyang Hospital of Wenzhou Medical University, Dongyang, Zhejiang, China; 6 Division of Urology, Taipei Hospital Renai Branch, Taipei, Taiwan; 7 Department of Urology, National Yang-Ming University School of Medicine, Taipei, Taiwan; 8 Department of Obstetrics and Gynaecology, Hsinchu MacKay Memorial Hospital, Hsinchu, Taiwan; 9 Department of Sports Medicine, College of Health Care, China Medical University, Taichung, Taiwan; 10 Department of Orthopedic Surgery, China Medical University Hospital, Taichung, Taiwan; 11 Department of Pharmacology, School of Medicine, China Medical University, Taichung, Taiwan; 12 Chinese Medicine Research Center, China Medical University, Taichung, Taiwan; 13 Department of General Surgery, Affiliated Dongyang Hospital of Wenzhou Medical University, Dongyang, Zhejiang, China

**Keywords:** S1P, PDGF-A, chondrosarcoma, angiogenesis, EPCs

## Abstract

The malignant bone tumors that are categorized as chondrosarcomas display a high potential for metastasis in late-stage disease. Higher-grade chondrosarcomas contain higher levels of expression of platelet-derived growth factor (PDGF) and its receptor. The phosphorylation of sphingosine by sphingosine kinase enzymes SphK1 and SphK2 generates sphingosine-1-phosphate (S1P), which inhibits human chondrosarcoma cell migration, while SphK1 overexpression suppresses lung metastasis of chondrosarcoma. We sought to determine whether S1P mediates levels of PDGF-A expression and angiogenesis in chondrosarcoma. Surprisingly, our investigations found that treatment of chondrosarcoma cells with S1P and transfecting them with SphK1 cDNA increased PDGF-A expression and induced angiogenesis of endothelial progenitor cells (EPCs). Ras, Raf, MEK, ERK and AP-1 inhibitors and their small interfering RNAs (siRNAs) inhibited S1P-induced PDGF-A expression and EPC angiogenesis. Our results indicate that S1P promotes the expression of PDGF-A in chondrosarcoma via the Ras/Raf/MEK/ERK/AP-1 signaling cascade and stimulates EPC angiogenesis.

## INTRODUCTION

The malignant bone tumors that constitute chondrosarcomas are not easy to diagnose or treat [1, 2], being characterized by poor responsiveness to conventional chemotherapy and radiotherapy [3], and high-grade chondrosarcoma has a poor prognosis with low survival rates despite wide surgical resection, which is considered to be the cornerstone of treatment [4].

Metastasis is the primary cause of cancer mortality [5–7]. Angiogenesis is vital for the development of cancer and metastasis [8–10]; the chief proangiogenic factor in these processes is vascular endothelial growth factor-A (VEGF-A) [11, 12]. Another crucial mediator of tumor angiogenesis and metastasis is the platelet-derived growth factor receptor (PDGFR); a positive correlation has been observed between levels of PDGFR-α expression and the aggressiveness of chondrosarcoma [13], so it is important to examine the molecular mechanisms underlying PDGF expression in human chondrosarcoma cells. Data are awaited from phase II trials investigating the efficacy of pazopanib, a potent PDGFR inhibitor, in different chondrosarcoma patient populations [14].

Sphingosine-1-phosphate (S1P), a platelet-derived lysophospholipid mediator, strongly inhibits PDGR-induced chemotaxis and cellular Rac activity in vascular smooth muscle cells [15]. Interestingly, deleting the S1P_2_ receptor promotes murine embryonic fibroblast migration towards S1P and also PDGF, which stimulates S1P production; S1P_2_ deletion also increases the enzymatic expression and activity of sphingosine kinase 1 (SphK1), which is responsible for producing S1P [16]. S1P/S1P receptor signaling has been discussed to regulate angiogenesis and vasculogenesis [17, 18]. S1P is known to regulate various cellular processes that are involved in cancer: SphK1 maintains tumor cell survival and promotes the progression of hormone-independent prostate and breast cancer [19, 20]; SphK1 overexpression stimulates Ras-dependent mechanisms that transform fibroblasts into fibrosarcoma; in estrogen receptor-positive breast cancer, high tumoral SphK1 expression is associated with poorer survival and shorter times to disease recurrence; and S1P promotes tumor neovascularization and induces inflammation involved in cancer progression [21]. Moreover, SphK1 and S1P encourage tumor growth and angiogenesis, metastasis and apoptotic resistance [22]. On the contrary, S1P also inhibits cancer cell migration by activation of S1P_2_- and ROCK-mediated vimentin S71 phosphorylation [23]. S1P is also capable of inhibiting endothelial cell angiogenesis [24], and of suppressing cell proliferation by inactivating Akt in keratinocytes [25] and prostate cancer cells [19]. We have previously demonstrated that S1P inhibits the migration of human chondrosarcoma cells and that SphK1 overexpression decreases metastasis to the lungs in a chondrosarcoma xenograft model [26]. Here, we sought to elucidate the relationship between S1P, PDGF-A expression and tumor angiogenesis, as well as characterize the molecular process whereby S1P induces PDGF-A-dependent angiogenesis in human chondrosarcoma cells.

## RESULTS

### S1P enhances PDGF-A-dependent EPCs angiogenesis

**Figure 1:**
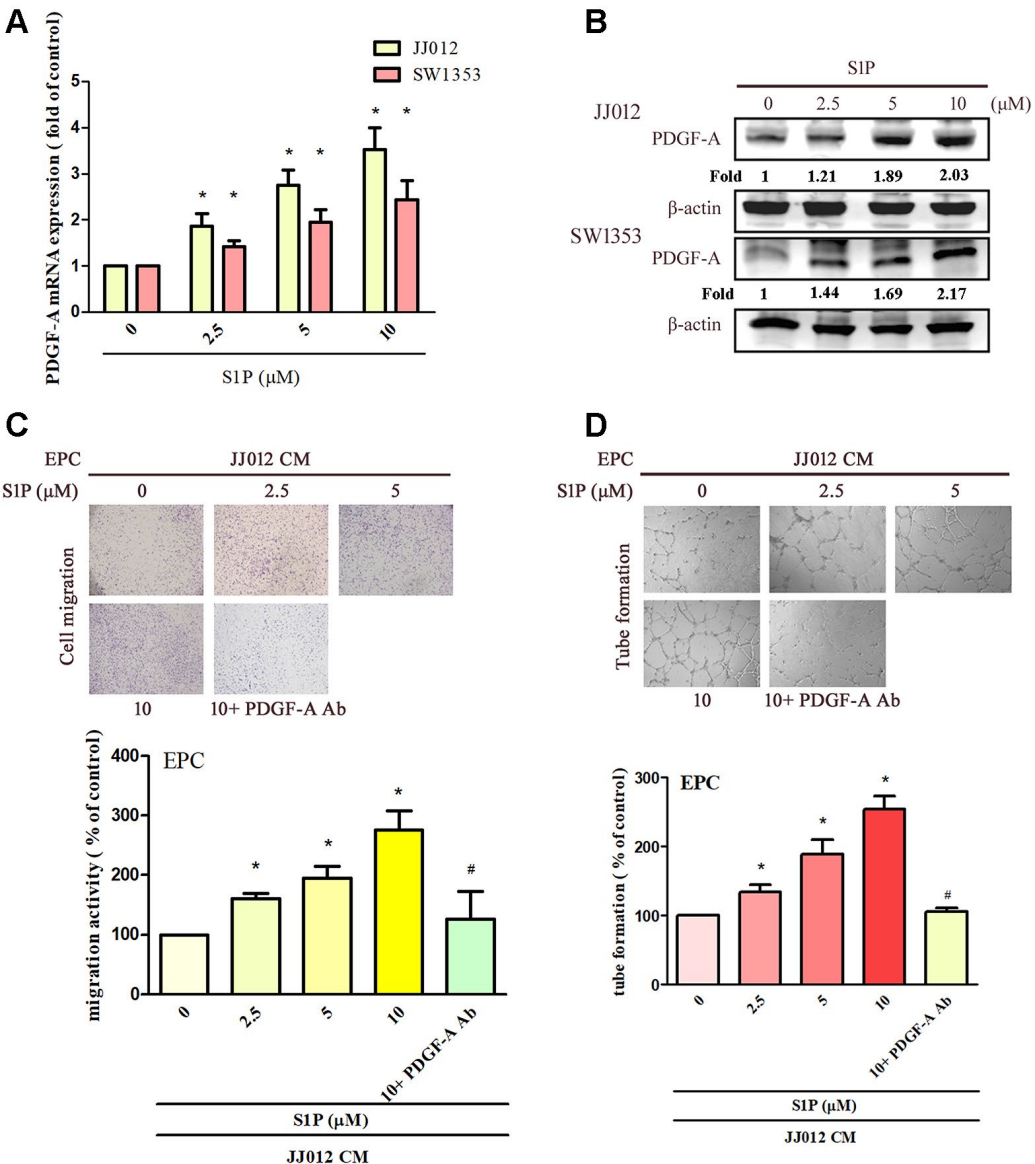
**S1P increases PDGF-A expression and angiogenesis in human chondrosarcoma cells.** (**A**, **B**) Chondrosarcoma cells were incubated with S1P (2.5–10 μM) for 24 h; PDGF-A expression was examined by qPCR and Western blot assays (n=4). (**C**, **D**) Chondrosarcoma cells were incubated with S1P for 24 h then stimulated with PDGF-A or IgG antibody (1 μg/ml) for 30 min. The conditioned medium (CM) was then collected and applied to endothelial progenitor cells (EPCs) (n=4). EPC migration and tube formation was measured. Results are expressed as the mean ± SEM. *p < 0.05 as compared with the control group; #*p* < 0.05 as compared with the S1P-treated group.

**Figure 2:**
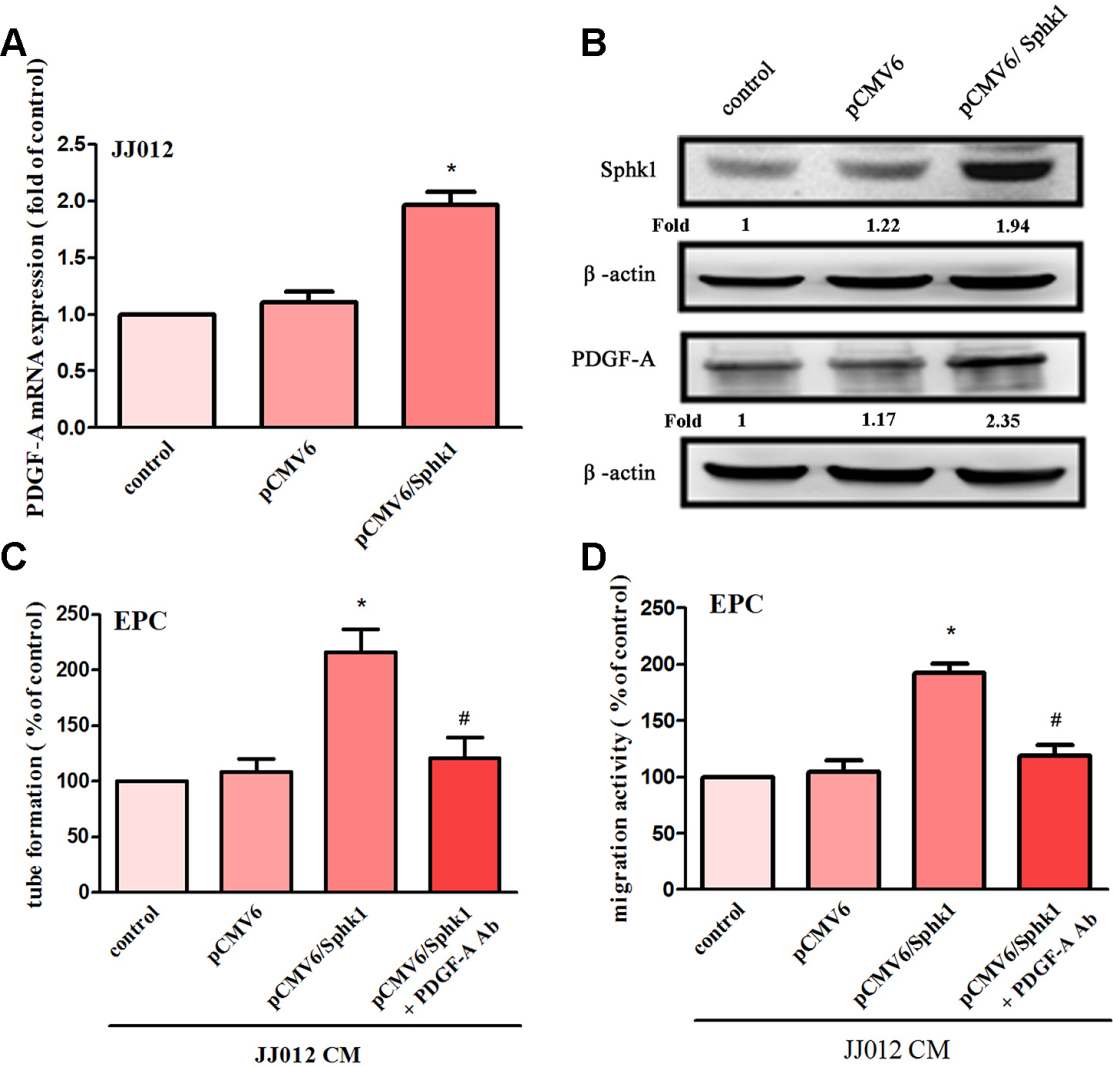
**Overexpression of SphK1 facilitates in PDGF-A expression and angiogenesis in human chondrosarcoma.** (**A**, **B**) Chondrosarcoma cells were transfected with SphK1 cDNA; SphK1 and PDGF-A expression was examined by qPCR and Western blot assays (n=5). (**C**, **D**) The CM was applied to EPCs and analyses assessed migratory and tube formation activity (n=4). Results are expressed as the mean ± SEM. *p < 0.05 as compared with the vector group.

The application of S1P to chondrosarcoma cell lines JJ012 and SW1353 concentration-dependently augmented PDGF-A mRNA and protein expression (Figure 1A, 1B). Evaluation of S1P-regulated angiogenesis in chondrosarcoma cells by EPC migration and tube formation assays [27] revealed that conditioned medium (CM) from S1P-treated chondrosarcoma cells stimulated EPC migration and tube formation (Figure 1C, 1D), whereas PDGF-A monoclonal antibody (mAb) treatment suppressed these events (Figure 1C, 1D). To confirm the role of S1P in EPC angiogenesis, the cells were transfected with SphK1 cDNA. We observed that overexpression of SphK1 cDNA increased levels of SphK1 protein expression (Figure 2B). Overexpression of SphK1 also increased PDGF-A mRNA and protein expression, EPC migration and tube formation (Figure 2). In addition, the PDGF-A mAb also blocked SphK1 facilitated EPC migration and tube formation (Figure 2C, 2D). Thus, S1P appears to stimulate EPC angiogenesis in a PDGF-A-dependent manner.

### S1P promotes PDGF-A-mediated angiogenesis through the Ras/Raf/MEK/ERK pathway

**Figure 3:**
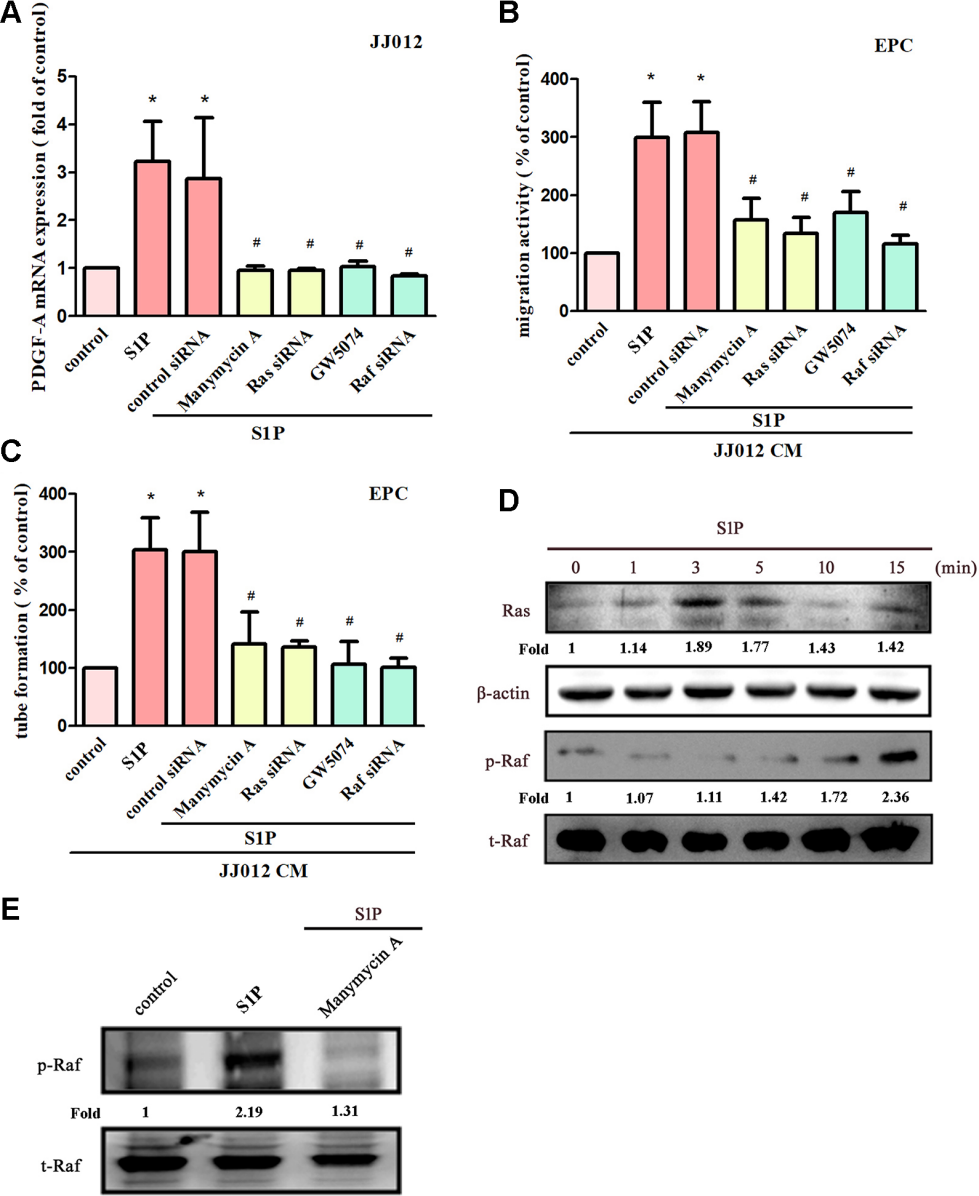
**The Ras and Raf pathways mediate S1P-promoted PDGF-A expression and angiogenesis.** (**A**) Cells were pretreated for 30 min with manumycin A (10 μM) and GW5074 (10 μM), or transfected with Ras and Raf siRNAs then stimulated with S1P (10 μM). PDGF-A expression was examined by qPCR assays (n=5). (**B**, **C**) The CM was applied to EPCs and analyses assessed migratory and tube formation activity (n=4). (**D**) JJ012 cells were incubated with S1P; Ras and Raf activity was examined by Western blot assay (n=3). (**E**) JJ012 cells were pretreated with manumycin A for 30 min, then stimulated with S1P and Raf phosphorylation was examined (n=3). Results are expressed as the mean ± SEM. *p < 0.05 as compared with the control group; #*p* < 0.05 as compared with the S1P-treated group.

The Ras/Raf/MEK/ERK signaling pathway regulates tumor angiogenesis and metastasis [28, 29]. Treatment of cells with manumycin A (a Ras inhibitor) or GW5074 (a Raf inhibitor) suppressed S1P-enhanced PDGF-A expression, EPC migration and tube formation (Figure 3A–3C). Next, Ras and Raf siRNAs were used to confirm the results obtained from pharmacological inhibitors. We found that Ras and Raf siRNAs abolished S1P-mediated effects (Figure 3A–3C). Incubation of chondrosarcoma cells with S1P enhanced Ras kinase activity and Raf phosphorylation (Figure 3D). The Ras inhibitor also reduced S1P-enhanced phosphorylation of Raf (Figure 3E), indicating that Ras serves as an upstream molecule of Raf.

**Figure 4:**
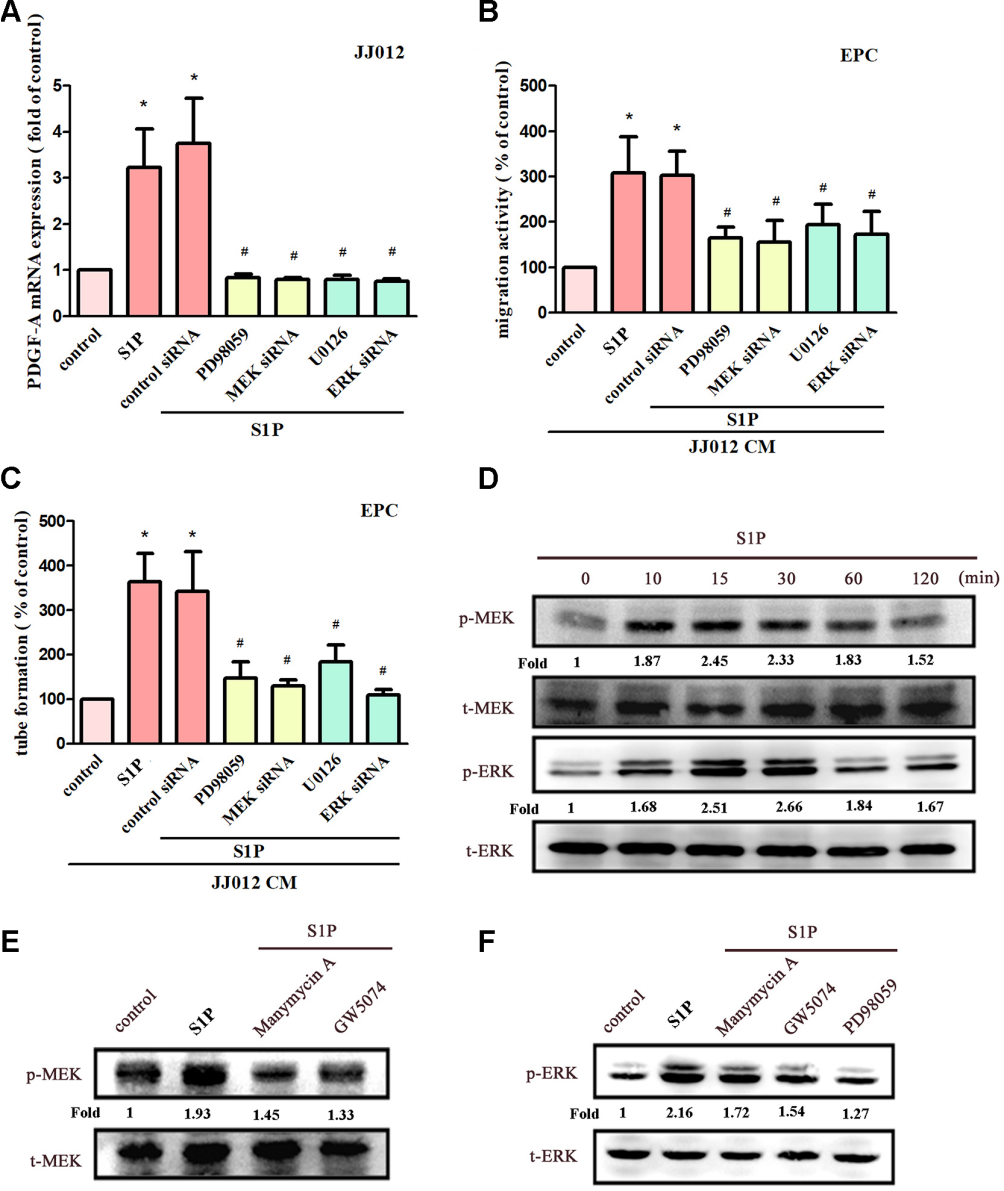
**The MEK and ERK pathways mediated S1P-promoted PDGF-A expression and angiogenesis.** (**A**) Cells were pretreated for 30 min with PD98059 (10 μM) and U0126 (5 μM), or transfected with MEK and ERK siRNAs, then stimulated with S1P (10 μM). PDGF-A expression was examined by qPCR assays (n=5). (**B**, **C**) The CM was applied to EPCs and analyses assessed migratory and tube formation activity (n=4). (**D**) JJ012 cells were incubated with S1P; MEK and ERK phosphorylation was examined by Western blot assay (n=3). (**E**, **F**) JJ012 cells were pretreated with manumycin A, GW5074 and PD98059 for 30 min, then stimulated with S1P (10 μM). MEK and ERK phosphorylation was examined (n=3). Results are expressed as the mean ± SEM. *p < 0.05 as compared with the control group; #*p* < 0.05 as compared with the S1P-treated group.

MEK/ERK is a common downstream signaling pathway of Ras and Raf proteins [28, 30]. Incubating chondrosarcoma cells with MEK inhibitors (PD98059 and U0126) or siRNAs against MEK and ERK effectively reduced S1P-enhanced PDGF-A expression, EPC migration and tube formation (Figure 4A–4C). Stimulation of chondrosarcoma cells by S1P promoted MEK and ERK phosphorylation (Figure 4D). Conversely, S1P-induced phosphorylation of MEK and ERK was reduced when cells were pretreated with Ras, Raf and MEK inhibitors (Figure 4E, 4F). These results suggest that S1P acts via the Ras/Raf/MEK/ERK signaling mechanism to enhance levels of PDGF-A expression and angiogenic activity in human chondrosarcoma cells.

### AP-1 transcriptional activity regulates S1P-promoted PDGF-A expression and angiogenesis

**Figure 5:**
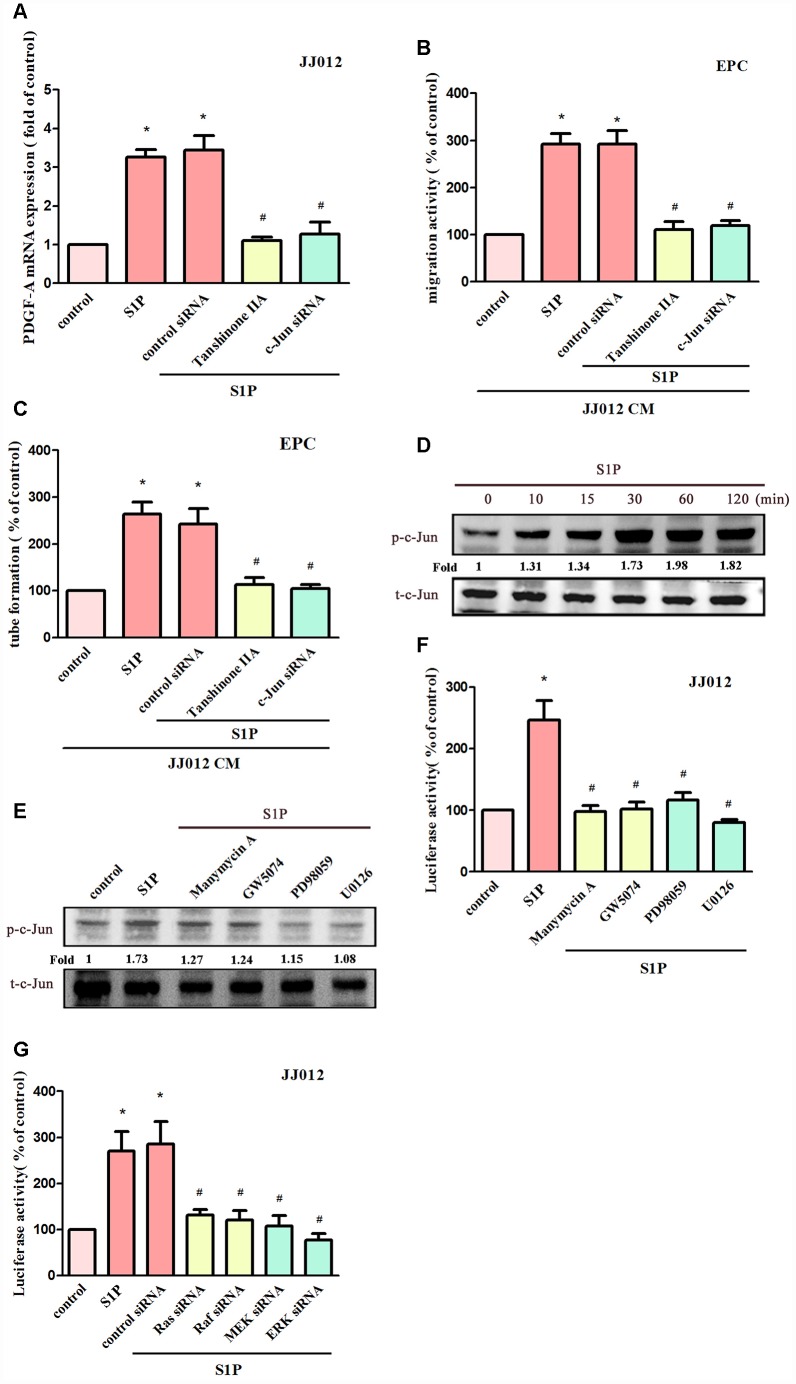
**AP-1 is involved in S1P-facilitated PDGF-A expression and angiogenesis.** (**A**) Cells were pretreated for 30 min with tanshinone IIA (3 μM), or transfected with c-Jun siRNA, then stimulated with S1P (10 μM). PDGF-A expression was examined by qPCR assays (n=5). (**B**, **C**) The CM was applied to EPCs and analyses assessed migratory and tube formation activity (n=4). (D) JJ012 cells were incubated with S1P (10 μM); c-Jun phosphorylation was examined by Western blot assay (n=3). (**E**) JJ012 cells were pretreated with manumycin A, GW5074, PD98059 and U0126 for 30 min, then stimulated with S1P (10 μM). The c-Jun phosphorylation was examined (n=3). (**F**, **G**) JJ012 cells were pretreated with Ras, Raf, MEK and ERK inhibitors or siRNAs, then stimulated with S1P (10 μM) and AP-1 Luciferase activity was examined (n=4). Results are expressed as the mean ± SEM. *p < 0.05 as compared with the control group; #*p* < 0.05 as compared with the S1P-treated group.

AP-1 appears to regulate PDGF gene expression [31]. We therefore examined whether AP-1 influences S1P-mediated PDGF-A expression in chondrosarcoma cells. Transfecting cells with an AP-1 inhibitor (tanshinone IIA) or c-Jun siRNA reduced S1P-promoted PDGF-A expression (Figure 5A); these compounds also suppressed S1P-induced EPC migration and tube formation (Figure 5B, 5C). S1P significantly promoted c-Jun phosphorylation (Figure 5D), which was reduced by pretreatment with Ras, Raf and MEK inhibitors (Figure 5E). To confirm that the Ras/Raf-1/MEK1/ERK signaling pathway mediated S1P-enhanced activation of AP-1, the AP-1 luciferase promoter plasmid was used. Treatment of cells with S1P augmented AP-1 luciferase activity, while pretreating the cells with Ras, Raf, MEK and ERK inhibitors or their siRNAs reduced S1P-induced AP-1 luciferase activity (Figure 5F, 5G). Activation of Ras, Raf-1, MEK1 and ERK appears to be involved in S1P-induced AP-1 activation in human chondrosarcoma cells.

## DISCUSSION

Chondrosarcomas are heterogeneous, malignant bone neoplasms [26, 32] that are characterized by an increasing propensity for metastasis at higher pathological grades. Chemotherapy and radiotherapy are of limited effectiveness in chondrosarcoma; surgery is the favored therapeutic option [33]. Growth rates of many low- and moderate-grade chondrosarcomas are relatively slow; approximately 15% of all deaths due to metastasis occur more than 5 years after diagnosis [34]. This phenomenon offers a window of opportunity for the therapeutic prevention of chondrosarcoma metastasis. Our previous investigation reported that S1P inhibits migration, invasion and metastasis in human chondrosarcoma [26]. Here, we found that S1P enhanced PDGF-A expression in human chondrosarcoma and facilitated EPC angiogenesis through the Ras, Raf, MEK, ERK and AP-1 signaling pathways. The human umbilical vein endothelial cell (HUVEC) are common used model of tumor angiogenesis. However, few reports used this model to examine the angiogenic effect in chondrosarcoma. Whether EPCs or HUVEC are best model to study in the context of chondrosarcoma needs further study.

Human chondrosarcoma cell lines show upregulated PDGF and PDGFR activities, which are required for tumor growth and metastasis [35]. Pazopanib (a PDGFR inhibitor) was associated with clinical benefits in a patient with metastatic chondrosarcoma that had failed to respond to first-line chemotherapy [14]. Pazopanib has also demonstrated efficacy (prolonged disease stabilization for over 6 months) and good tolerability in 8 patients with progressive chondrosarcoma administered pazopanib 800 mg/day [36]. The PDGF/PDGFR axis is therefore a promising target for chondrosarcoma progression and metastasis. This paper reports that S1P enhanced PDGF-A mRNA and protein expression in both chondrosarcoma cell lines. Overexpression of SphK1 facilitated PDGF-A production. Otherwise, S1P also increases other angiogenic factors expression in chondrosarcoma, the PDGF-A is most upregulated (Supplementary Figure 1). Importantly, incubating EPCs with PDGF-A mAb antagonized S1P-induced migration and tube formation, indicating that S1P enhances EPC angiogenesis via a PDGF-A-dependent manner. Treatment of EPC with S1P slightly increased EPC tube formation (Supplementary Figure 2), indicating S1P also have direct effect in EPC angiogenesis.

S1P, a simple bioactive sphingolipid, is generated by SphK-induced phosphorylation of sphingosine. S1P regulates cancer-related processes such as autophagy, proliferation, angiogenesis and migration, by binding to its membranous receptors or by targeting the intracellular molecules [19, 22]. SphK1 upregulation has been identified in several cancers and is associated with poor survival prognosis [37]. We have previously demonstrated that S1P inhibits cellular migratory activity in human chondrosarcoma [26]. However, this investigation has demonstrated the opposite effect, in that S1P enhanced PDGF-A expression and EPC angiogenesis in human chondrosarcoma cell lines. We speculate that these apparently contradictory results may be explained in several ways. Firstly, angiogenesis is observed in the early stages of tumorigenesis, whereas endothelial cell migration generally occurs during tumor metastasis [38]. Secondly, EPC angiogenesis is only part of tumor angiogenesis; the endothelium cells may play a more important role than EPCs during tumor angiogenesis. Thirdly, the five S1P receptors may mediate different cell functions [16]. Finally, our investigation did not include an *in vivo* animal model to confirm the mediatory effects of S1P upon EPC angiogenesis.

The activation of the Ras/Raf/MEK/ERK signaling pathway is essential in many types of cancer for mediating multiple cellular functions, including cell survival, proliferation, migration and autophagy [29]. The Ras/Raf/MEK/ERK signaling pathway is also associated with tumor angiogenesis, as is seen with toluhydroquinone (2-methyl-1,4-hydroquinone), a marine-derived fungi, which reduces HUVEC angiogenesis via the Ras/Raf/MEK/ERK cascade [39]. In this study, we observed that S1P enhanced Ras, Raf, MEK and ERK activation, while Ras, Raf, MEK and ERK inhibitors and their siRNAs inhibit S1P-induced PDGF-A expression. It appears that the Ras/Raf/MEK/ERK signaling pathway is involved in S1P-mediated PDGF-A expression and angiogenesis. AP-1 has been indicated controls PDGF expression [31]. In current study, we also confirm AP-1 inhibitor or c-Jun siRNA abolished S1P-promoted PDGF-A expression. Otherwise, KLF5 has been previously shown to regulate PDGF-A expression through HIF1α [40]. Whether HIF1α also involve S1P-controled PDGF-A production are needs further examination.

**Figure 6:**
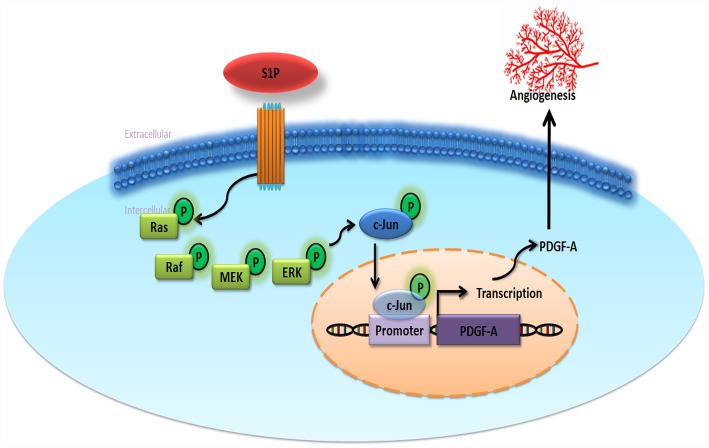
**Schematic diagram summarizes the mechanisms of S1P-promoted tumor angiogenesis in chondrosarcoma.** S1P facilitates PDGF-A production via the Ras/Raf/MEK/ERK/AP-1 signaling pathway in human chondrosarcoma cells and subsequently induces EPC angiogenesis.

The very poor prognosis for chondrosarcoma with metastatic disease makes it imperative that we can effectively prevent metastasis [41]. Our study shows that S1P enhances EPC angiogenesis in human chondrosarcoma, as a result of the upregulation of PDGF-A expression through the Ras/Raf/MEK/ERK/AP-1 signaling pathway (Figure 6). Clearly, therapeutic interventions are needed that focus on the functioning of the S1P signaling pathway and its relationship with PDGF-A in chondrosarcoma.

## MATERIALS AND METHODS

### Materials

S1P was obtained from Avanti Polar Lipid Inc. (Alabaster, AL, USA). We obtained SphK1 (GTX33516) antibody from Genetex (Irvine, CA, USA), Ras (SC-520), Raf (SC-133), MEK (SC-6250), ERK (SC-1647), PDGF-A (SC-9974), c-Jun (SC-74543), p-Raf (SC-101791), p-ERK (SC-7383) and p-c-Jun (SC-822) from Santa Cruz (Santa Cruz, CA, USA), and p-MEK (2338S) from Cell Signaling Technology (Danvers, MA, USA). ON-TARGETplus siRNAs were purchased from Dharmacon Research (Lafayette, CO, USA). SphK1 cDNA clone plasmid was purchased from OriGene (Rockville, MD, USA). Gibco-BRL Life Technologies (Grand Island, NY, USA) supplied fetal bovine serum (FBS), DMEM, α-MEM, and all other cell culture reagents. Promega (Madison, WI, USA) supplied the pSV-β-galactosidase vector and luciferase assay kits. The AP-1 luciferase plasmid was purchased from Stratagene (La Jolla, CA). All other chemicals or inhibitors were purchased from Sigma-Aldrich (St. Louis, MO, USA).

### Cell culture

The human chondrosarcoma cell line JJ012 was kindly supplied by Dr. Sean P. Scully’s laboratory at the University of Miami School of Medicine (Miami, FL, USA). The human chondrosarcoma cell line SW1353 was purchased from the American Type Culture Collection (Manassas, VA, USA). Chondrosarcoma cell culture conditions were recorded as previously described [42]. Human EPCs were isolated and cultured by a standard method as previously described [43, 44]. This study was approved by the Institutional Review Board of Mackay Medical College, New Taipei City, Taiwan (P1000002).

### Preparation of conditioned medium (CM)

Human chondrosarcoma cells were plated in 6-well plates at a density of 2×10^5^ cells/well in culture medium, grown to 80% confluence. Human chondrosarcoma cells were pretreated with pharmacological inhibitors, or transfected with siRNA followed by stimulation with S1P for 24 h. After treatment, cells were washed and changed to serum-free medium. CM was then collected 2 days after the change of medium and stored at −80°C until use.

### EPC tube formation assay

The tube formation assay was performed using Matrigel-coated (BD Biosciences, Bedford, MA, USA) 48-well plates. EPCs were resuspended at a density of 2 × 10^4^/200 μL in culture medium (50% EGM-MV2 medium and 50% chondrosarcoma cell CM) and added to the wells. Measurement of tube formation examined the differentiation and formation of capillary-like tubules on EPCs, according to previously described procedures [45, 46].

### EPC migration assay

Transwell inserts (8-μm pore size; Costar, NY, USA) were used for migration determination. Approximately 5 × 10^3^ cells were added to the upper chamber in 200 μL of 10% FBS MV2 complete medium. The lower chamber contained 150 μL 20% FBS MV2 complete medium and 150 μl CM. EPC migratory ability was assayed using the method from our previous works [45, 47].

### Western blot analysis

Chondrosarcoma cells were seeded on 6-well plates at a density of 3×10^5^ cells/well, grown to 80% confluence and then handle different conditions according to experimental needs the next day. Cell lysates underwent electrophoresis with SDS-PAGE and were transferred to PVDF membranes according to the method described in our previous studies [48, 49]. After blocking the blots with 4% bovine serum albumin, the blots were treated with primary antibody and then peroxidase-conjugated secondary antibody. Visualizations of the blots were accomplished by enhanced chemiluminescence using the UVP Biospectrum system (UVP, Upland, CA, USA).

### Quantitative real-time PCR (qPCR) of mRNA

Human chondrosarcoma cells were plated in 6-well plates at a density of 2×10^5^ cells/well in culture medium, grown to 80% confluence and then handle different conditions according to experimental needs the next day. Total RNA was extracted from chondrosarcoma cells using TRIzol reagent. qPCR analysis was conducted according to an established protocol [50, 51].

### Ras kinase activity

Chondrosarcoma cells were seeded on 6-well plates at a density of 3×10^5^ cells/well, grown to 80% confluence. Cells were treated with S1P then the activation of Ras (Ras-GTP) was detected using the Ras-binding domain of Raf-1 to pull down active Ras (Ras Activation Assay Kit; Millipore, CA, USA), according to the manufacturer's recommendations. Following separation by SDS-PAGE, proteins were transferred to membranes that were probed with an anti-RAS antibody [34].

### siRNA transient transfection and luciferase reporter assay

Chondrosarcoma cells were seeded on 12-well plates at a density of 1×10^5^ cells/well, grown to 80% confluence and transfected the next day. Cells were co-transfected with 0.8 μg AP-1-luciferase reporter gene construct and 0.4 μg β-galactosidase using Lipofectamine 2000, as per the manufacturer's instructions. After 24 h of transfection, the cells were exposed to S1P. Luciferase activity was determined using the luciferase assay kit [52, 53].

### Statistical analysis

All data are presented as means ± standard errors of the means (SEMs) of at least three independent experiments. The Student’s t-test determined statistical differences between samples and the Bonferroni post hoc procedure was performed for a one-way analysis of variance (ANOVA) of statistical comparisons between more than two samples, and p-values of less than 0.05 were considered significant.

## Supplementary Material

Supplementary Figures
