# Supplementary material for: Sphingosine-1-phosphate promotes PDGF-dependent endothelial progenitor cell angiogenesis in human chondrosarcoma cells
Source: Aging (Albany NY). 2019 Dec 6;11(23):11040–53. doi: 10.18632/aging.102508 (PMC6932882; doi:10.18632/aging.102508)
Supplement: Supplementary Figures [file aging-11-102508-s001..pdf]

SUPPLEMENTARY FIGURES

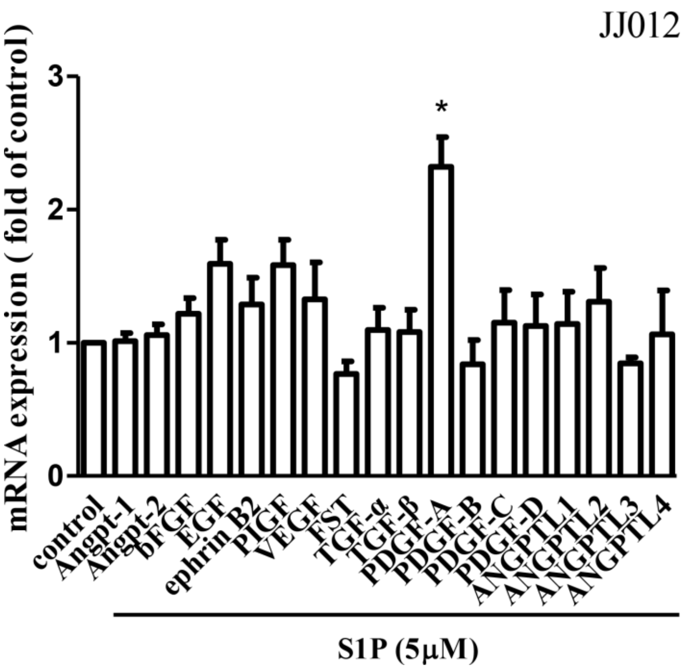

Supplementary Figure 1. S1P promotes angiogenic factors expression in human chondrosarcoma cells. Chondrosarcoma cells were incubated with S1P (10 μM) for 24 h; mRNAs expression was examined by qPCR.

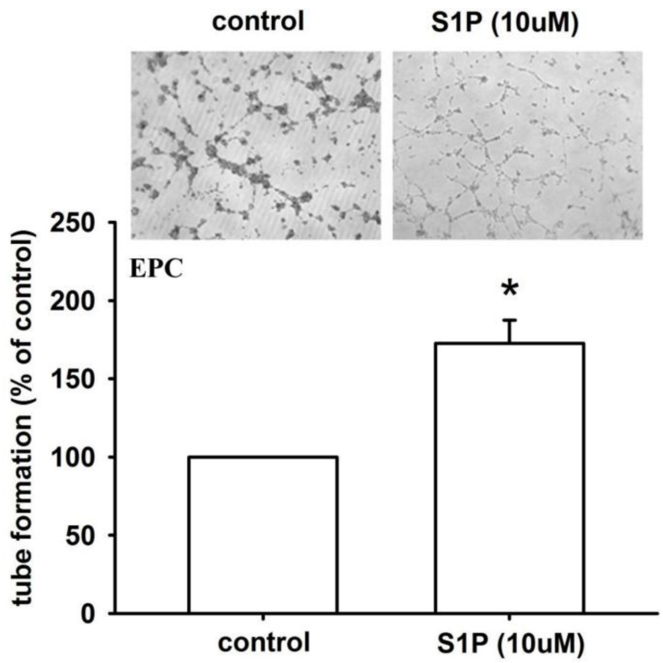

Supplementary Figure 2. S1P increases EPC tube formation. EPCs were incubated with S1P (10 μM) for 24 h; EPC tube formation was measured.
